# Ruptured ectopic pregnancy as complete hydatidiform mole: Case report and review of the literature

**DOI:** 10.3389/fsurg.2022.1036435

**Published:** 2022-11-02

**Authors:** Aikaterini Athanasiou, Anis Féki, Arrigo Fruscalzo, Benedetta Guani, Nordine Ben Ali

**Affiliations:** Department of Obstetrics and Gynecology, Fribourg Cantonal Hospital Chemin des Pensionnats, Villarssur-Glâne, Switzerland

**Keywords:** haemoperitoneum, mole, ectopic pregnancy, tubal mass, adnexal mass

## Abstract

Usually, a hydatidiform mole (HM) develops inside the uterus. The occurrence of HM in ruptured tubal pregnancy is exceptional. Cases reported in the literature are scarce. In this article, a case of haemoperitoneum secondary to a ruptured fallopian tube by a complete mole is reported. A 50-year-old gravida 2, para 1 was admitted to the emergency department for acute abdominal pain. After the clinical examination, an abdominal sonography and CT scan were done, revealing the presence of an adnexal left mass associated with an important haemoperitoneum. A urine pregnancy test was done and was positive, indicating an immediate laparoscopic exploration. The laparoscopy revealed a haemoperitoneum secondary to a ruptured tubal mass. The pathological exam concluded a complete hydatidiform mole (CHM) invading the wall of the fallopian tube. Any acute abdominal pain in a potentially pregnant woman imposes first the routine realization of a pregnancy test. The occurrence of CHM in a ruptured fallopian tube is particularly rare and has exceptionally been diagnosed before the laparoscopic exploration.

## Introduction

Gestational trophoblastic diseases (GTD) belong to a group of both benign and malignant tumors that are formed from placental tissue and include the following four formations: Placental site trophoblastic tumor, choriocarcinoma, epithelioid trophoblastic tumor, and hydatidiform mole (complete and partial) (1). The hypernym GTD is a broad term for a range of pregnancy related problems that develop due to irregular multiplication of the trophoblasts and swelling of the placental villi (2). GTD results from genetic disorders that can lead to placental and trophoblastic deformities (1).

Ectopic molar pregnancy is rare and has been reported in the fallopian tube (3), in the cervical canal (4), in the uterine cornua (5) and even in the ovary (6). The heterotopic pregnancy phenomenon also concerns the molar disorder with cases of complete molar intrauterine pregnancy (7), or normal intrauterine pregnancy and a corneal or tubal complete mole (8).

## Case report

### History and examination

A 50-year-old gravida 2, para 1 was brought by ambulance to the general emergency department of our hospital. She reported in her history a vaginal delivery and being followed for Crohn’s disease that did not require medication for the moment. She still had periods but they were irregular and she did not use any contraception.

Clinical examination revealed a distended abdomen with rebound and a maximum site of pain in the left iliac fossa. There was no abdominal guarding or rigidity. The vaginal examination revealed mild endocervical non-active bleeding. The patient was unable to provide urine on admission, explaining why the routine urine human chorionic gonadotropin (Beta hCG) test was not done before the imaging examinations.

**Figure 1:**
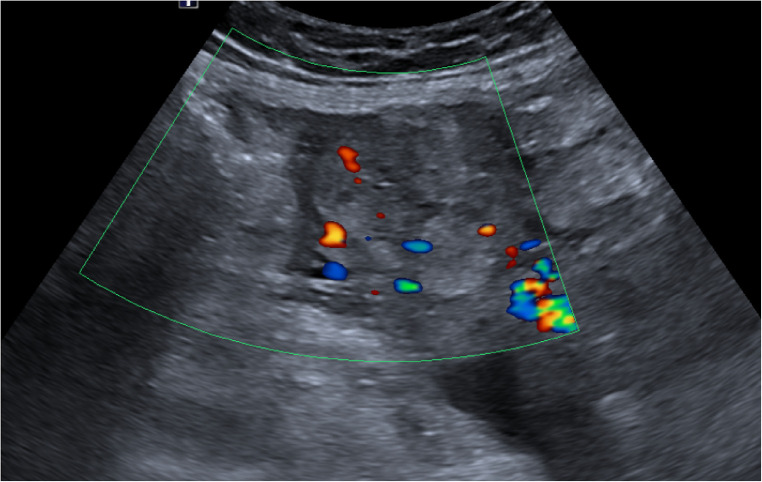
Ultrasonographic appearance of the left adnexal mass: scarce colour Doppler flow in the left adnexal mass. The effectiveness of color-flow Doppler in the setting of a molar pregnancy is considered debatable. The typical finding of a molar pregnancy are the multiple small vesicles in a hyperechoic mass which classically give a “snowstorm” appearance.

**Figure 2:**
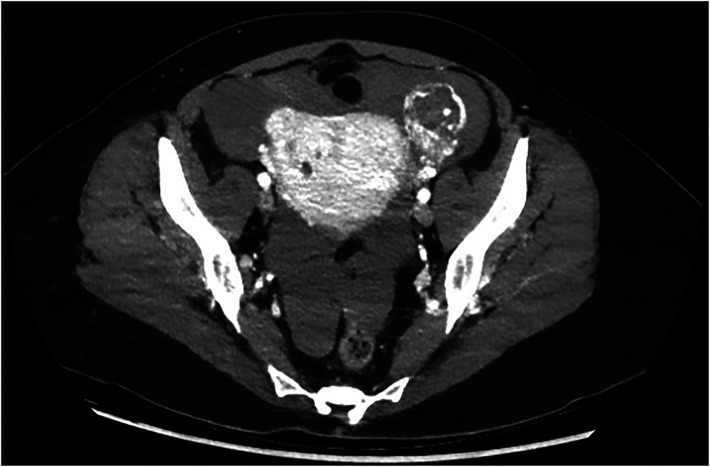
Ct scan: after intravenous injection of iobitridol (iodinated contrast agent), the CT scan showed the left adnexal mass with areas of low attenuation internally, outlined by highly enhanced contours.

Abdominal ultrasonography showed a left adnexal mass measuring 67 × 25 mm adjacent to the left ovary presenting a scarce colour doppler flow (Figure 1). An abundant haemoperitoneum was noted in the parieto-colic spaces and in Morison’s pouch. Haemodynamically, the patient was stable with a pulse of 80 beats/min and a blood pressure of 120/70 mmHg. Nevertheless, the skin examination noted a pallor. To complete the ultrasonography, an abdominal CT was realized, confirming the ultrasonographic observations (Figure 2). Routine haematological and biochemical examinations were obtained a second time: Beta hCG was 83,346 U/L and haemoglobin concentration was 7.7 gr/dl.

**Figure 3:**
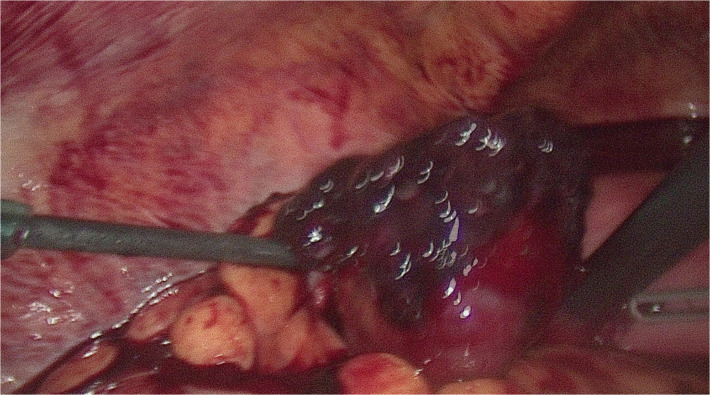
Intraoperative image of left fallopian tube ruptured by tissue with hydropic modifications which appear as semi-transparent tiny glistening cysts of variable size on its surface. A salpingectomy was performed due to the rupture of the tube.

Explorative laparoscopy was immediately performed and found a ruptured left tubal mass measuring 6 × 4 cm with a 1.5 L haemoperitoneum. Observation of the surface of the mass revealed tiny glistening cysts (Figure 3). Left salpingectomy was done and sent for histopathological evaluation.

### Histopathological findings

**Figure 4:**
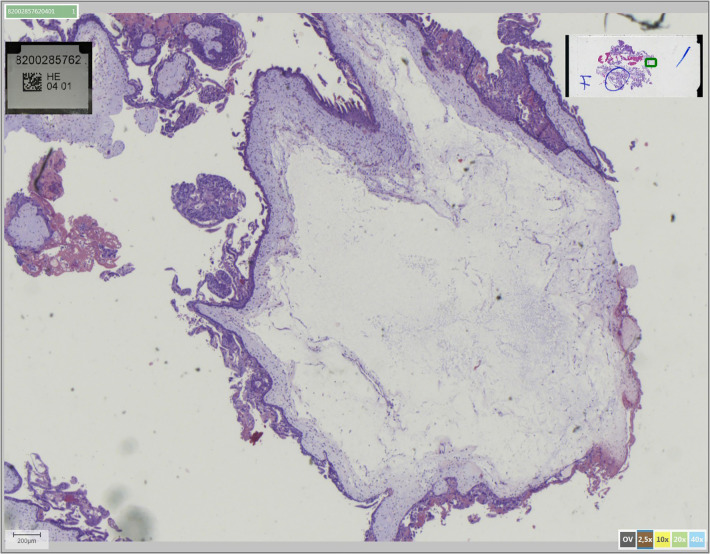
Pathology of the complete tubal hydatidiform mole: degeneration of chorionic villi around a central hydropic lacuna along with trophoblast development due to fertilization of the abnormal ovum defines the hydatidiform mole.

On histological examination, there were chorionic villi with circumferential trophoblastic proliferation, hydropic modification, scalloped villi and karyorrhexis (Figure 4). The immunohistochemical marker p57 was not expressed in the villus trophoblast or the stroma. It is an important diagnostic feature of complete hydatidiform mole and not pseudohydatidiform mole often described in tubal ectopic pregnancy (7, 8). The p57 marker is a protein that is formed from the imprinting of a paternal gene that expresses the maternal allele. Classically, complete hydatidiform moles (CHM) do not express this marker due to its androgenetic constitution, contrary to partial hydatidiform moles (HM) and non-molar pregnancy expressing it diffusely (9-11).

### Post-operative course

The post-operative period was uneventful, with return home on the first post-operative day. Because of the risk of a persistent trophoblastic disease, she was followed up by weekly serum Beta hCG until obtaining three titres less than 5 U/L and then monthly for 1 year. She was advised to use contraception and decided to use barrier methods.

## Discussion

We choose to present this case knowing that the support has not been optimal. Immediate unavailability of urine hCG likely led to an abdominal CT scan. A posteriori, we judge that this CT scan was performed unnecessarily and did not provide any additional information, leaving the final diagnosis of mole unsuspected.

### Epidemiology

Ectopic gestation is a common phenomenon with an incidence rate of 4.5–16.8/1,000 pregnancies (3). HM can be complete or incomplete and incidence is around 1/1,000 pregnancies and 3/1,000 pregnancies, respectively (9). The combination of an event of mole with an ectopic gestation is estimated to supervene in 1.5/1,000,000 pregnancies (12). More than 132 cases have been reported in the world literature (13). Burton et al. warn us that the incidence of tubal ectopic HM is not so evident and is usually over-diagnosed in histopathological specimens (14).

Risk factors that increase EP susceptibility are endometriosis, cesarean section, tubal surgery, pelvic inflammatory disease, smoking, fertility treatment, intrauterine devices and variant reproductive system anatomy (2). However, in about 50% of women with EP, the predisposing factors mentioned do not appear.

Ectopic molar pregnancy is rather scarce and this has prevented the determination of definite risk factors. In molar pregnancy, the principal risk factors are a history of GTD and higher maternal age (15). A retrospective study review by Al-Talib A, demonstrated that 63.7% of HM cases involved women older than 35 years old while 18.2% women younger than 20 years old (15).

### Radiological characteristics

If the patient is symptomatic, she will mostly present abdominal pain and/or vaginal bleeding. Before any complementary imagery exploration, the realization of a urine pregnancy test is fundamental to orient the diagnosis to involving a possible pregnancy. Ultrasonography by its availability and low cost is the first choice. Nevertheless, the characteristic Swiss cheese or snowstorm pattern is rarely encountered. In most cases, a specific heterogenous, hypoechoic solid mass with cystic spaces is observed. The diagnosis is sometimes suggested intraoperatively by the presence of glistening vesicles on the surface of the tumour.

One experience of more than 1,000 cases from a regional referral centre suggests that ultrasonography identifies less than 50% of intra uterine HM (16); but we can assume that our ultrasound equipment performs better than that used in this study, which dates from 2006.

### Diagnosis

HM is the consequence of a placental malformation due to genetic anomalies, producing cystic swelling and trophoblastic proliferation. In CHM, a diploid paternal genome is present after the fertilization of an empty ovum by a divided haploid spermatozoon. In a partial mole, a triploid genome is present after a dispermic fertilization of a haploid ovum. Beta hCG levels are not useful in differentiating between molar and non-molar ectopic pregnancies (17). Histopathological examination of the products of conception remains the current gold standard for diagnosis. Flow-cytometry DNA analysis is a useful complement for ploidy identification between diploid cells for complete mole and triploid cells for partial mole (18).

Sebire et al. compared molar products of conception from uterine curettage and fallopian tube pregnancies and described in some cases a greater degree of extra villous trophoblastic proliferation in ectopic gestation, inducing a risk of over-diagnosis (19).

### Treatment

In a haemodynamically stable patient, laparoscopy is preferable to laparotomy in view of the shorter hospital stay and faster convalescence. For reasons specific to our centre, we prefer salpingostomy to salpingectomy, knowing that in terms of fertility there is no significant gain in keeping the tube. In our case, the rupture of the fallopian tube justified the salpingectomy. In the case of an occult and unruptured ectopic pregnancy, we would probably have opted for a salpingostomy, with the risk of leaving molar material in the fallopian tube. Reading the literature, we find a case of partial HM successfully managed solely by salpingostomy. The question of a possible need for a second intervention to perform a salpingectomy vs. expectation remains open. It is important to note that the reported rate of rupture and haemoperitoneum is 67% in cases of ectopic molar pregnancy in comparison to 25%–30% in cases of non-molar ectopic pregnancy (20).

### Prognosis

It is assumed that the malignant potential of an ectopic molar pregnancy is similar to that of an intrauterine molar pregnancy (21). The risk of persistent trophoblastic disease is approximately 0.5% for partial mole and 15% for complete mole (22). Following up the good decrease of Beta hCG levels then maintenance of a negative hCG level are essential before declaring the patient cured. Govender et al. reported a case of pulmonary metastasis in a patient with mole of the fallopian tube coexisting with intrauterine pregnancy (23). In French-speaking Switzerland, a reference centre for molar pregnancy reviews the histological sections and makes recommendations for further treatment. Throughout the post-operative follow-up period, effective contraception is recommended.

Regarding future fertility, a meta-analysis by Capozzi et al., indicates that there is a significant difference in the rate of live births between complete and partial moles. Patients with complete moles had an increased live birth rate compared to patients after a partial mole pregnancy (24).

However, the occurrence of stillbirths, ectopic pregnancies, preterm birth and miscarriages didn’t show any significant difference between the two groups (24). The aforementioned study is helpful for clinicians in guiding patients who wish to conceive after a molar pregnancy (24).

## Conclusion

Clinical examination combined with ultrasound performs poorly in the preoperative detection of intrauterine or extrauterine moles. Histopathological examination remains the gold standard for making the diagnosis of mole for any product of conception.

## Data Availability

The original contributions presented in the study are included in the article/Supplementary Material, further inquiries can be directed to the corresponding author/s.
